# Seroconversions to Rickettsiae in US Military Personnel in South Korea

**DOI:** 10.3201/eid2106.141487

**Published:** 2015-06

**Authors:** Ju Jiang, Todd E. Myers, Patrick J. Rozmajzl, Paul C.F. Graf, Jean-Paul Chretien, Joel C. Gaydos, Allen L. Richards

**Affiliations:** Naval Medical Research Center, Silver Spring, Maryland, USA (J. Jiang, T.E. Myers, P.J. Rozmajzl, P.C.F. Graf, A.L. Richards);; Armed Forces Health Surveillance Center, Silver Spring (J.-P. Chretien, J.C. Gaydos);; Uniformed Services University of the Health Sciences, Bethesda, Maryland, USA (A.L. Richards)

**Keywords:** rickettsiosis, scrub typhus, South Korea, US military, rickettsia, seroconversions, typhus group rickettsiae, spotted fever group rickettsiae, scrub typhus group orientiae

**To the Editor:** Infections with typhus group rickettsiae (TGR), spotted fever group rickettsiae (SFGR), and scrub typhus group orientiae (STGO) have been reported among persons in South Korea in increasing numbers over the past decade (*1*,*2*). During 2001–2011 in South Korea, 51,825 orientiae group infections were reported (mean incidence 9.95 cases/100,000 residents/year) (*2*). TGR (*Rickettsia typhi*), SFGR (*R. akari, R. japonica, R. monacensis,* and *R. felis*), and STGO (*Orientia tsutsugamusi*) have been identified in their arthropod vectors and reservoirs in northern provinces and at US military training facilities in South Korea (*3*–*5*).

Currently, little data exist on the risk for rickettsioses and scrub typhus for US military deployed to South Korea. Thus, a retrospective serologic investigation to determine the level of exposure to rickettsiae among 9,303 military personnel deployed to South Korea was conducted. The study used de-identified predeployment and postdeployment serum samples made available from the Department of Defense Serum Repository (*6*). The study group consisted of men in combat-related jobs at US military training sites and military installations in South Korea during 1990–1995 while on active duty continuously for >1 year. This study protocol was reviewed and approved by the Naval Medical Research Command Institutional Review Board in compliance with all applicable federal regulations governing the protection of human subjects. 

Age range of the 9,303 soldiers in the study group was 17–52 (median 24) years. Most (99.6%) were stationed in Dongducheon, Yongtaeri, and Seoul, located in the Gyeonggi and Gangwon provinces in northern South Korea. Primary military occupation specialties were infantryman (58.8%), fighting vehicle infantryman (22.4%), indirect fire infantryman (12.3%), and heavy anti-armor weapons infantryman (6.5%).

The soldiers’ postdeployment serum samples (n = 9,303) were screened at a dilution of 1:100 for IgG against TGR, SFGR, and STGO by using group-specific ELISA whole-cell antigen preparations from *R. typhi* Wilmington, *R. conorii* Morrocan, and a mixture of *O. tsutsugamushi* Karp, Kato, and Gilliam, respectively (*7*,*8*). TGR, SFGR, and STGO IgG ELISA titers (range 100–>6,400) were determined for screen-positive (net absorbance >0.500) postdeployment serum samples, and results were compared with matched predeployment serum samples. Samples with a net total absorbance >1.000 for serum dilutions 1:100, 1:400, 1:1,600, and 1:6,400 were considered titer positive. The inverse of the highest dilution of titer positive serum that produced a net absorbance >0.200 was determined to be the titer. Serum samples from laboratory animals infected with *R. felis* reacted specifically in the SFGR ELISA but not in the TGR ELISA (K.R. Macaluso and A.L. Richards, unpub. data); thus, any soldier infected with *R. felis* would have reacted in the SFGR but not the TGR ELISA.

The postdeployment seropositivity in US military personnel for antibodies against TGR, SFGR, and STGO at a titer ≥100 were 1.3% (117/9,249), 9.0% (805/8,918), and 0.5% (44/9,135), respectively (Figure). Seropositivity occurred for 10 (0.1%), 181 (2.0%), and 15 (0.2%) men who showed evidence of infection (seroconversion or 4-fold rise in antibody titer) with TGR, SFGR, and STGO, respectively, during their deployment to South Korea (Figure). The chance of a soldier having an infection with SFGR was significantly higher than the chance of having an infection with TGR or STGO (χ^2^ test, p<0.05) (analysis performed in SAS version 9.4; SAS Institute Inc., Cary, NC, USA). For personnel who seroconverted or had a 4-fold rise in titer to TGR, SFGR, or STGO, the age range was 19–49 (median 25) years, and job specialties were infantrymen (63.5%), fighting vehicle infantrymen (16.4%), indirect fire infantrymen (14.2%), and heavy anti-armor weapons infantrymen (5.9%).

**Figure:**
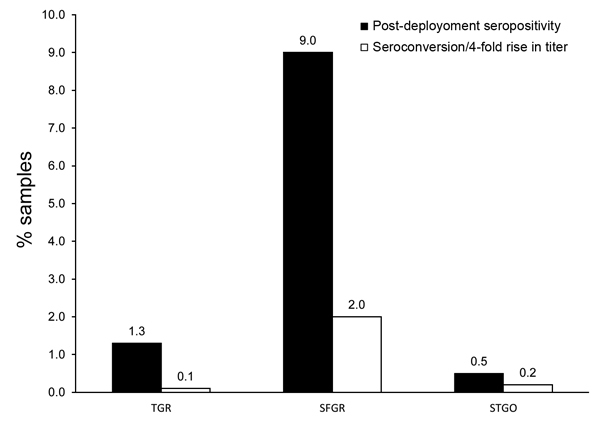
Evidence of rickettsiosis or scrub typhus among US military personnel deployed to South Korea. Black bars indicate postdeployment serum samples from US military personnel with a titer ≥1:100 (seropositive) to typhus group rickettsiae (TGR), spotted fever group rickettsiae (SFGR), or scrub typhus group orientia (STGO) IgG, as determined by ELISA. White bars indicate personnel determined by paired serum sample analyses to have seroconversion or 4-fold rise in titer between predeployment and postdeployment serum samples, indicating evidence of infection with the corresponding pathogen during deployment.

These results indicate that many US military personnel were exposed to rickettsiae and orientiae before their deployment to South Korea (Figure), perhaps because of previous deployments around the world or because of exposure to rickettsial agents at home (*8*–*10*). However, 206 (2.2%) of the men became infected with either a typhus group (n = 10) or spotted fever group (n = 181) rickettsia or a scrub typhus group orientia (n = 15) during their deployment to South Korea.

More SFGR infections occurred than TGR and STGO infections, although the pathogens for the latter infections (*R. typhi* and *O. tsutsugamushi*) are considered endemic to South Korea and are believed to affect the public and military health more than SFGR (*3*). The SFGR infections might correlate with recent observations of highly prevalent rickettsia-infected tick and *R. felis*–infected flea populations seen in South Korea (*4*,*5*). No evidence of co-infection was found in the men assessed during the deployment. These results suggest a risk for rickettsial disease, including scrub typhus and especially spotted fever, among US military personnel stationed in or visiting South Korea.
